# Molecular confirmation of the occurrence in Germany of *Anopheles daciae* (Diptera, Culicidae)

**DOI:** 10.1186/1756-3305-5-250

**Published:** 2012-11-12

**Authors:** Mandy Kronefeld, Marko Dittmann, Dorothee Zielke, Doreen Werner, Helge Kampen

**Affiliations:** 1 Friedrich-Loeffler-Institut, Federal Research Institute for Animal Health, Südufer 10, Greifswald – Insel Riems 17493, Germany; 2 Leibniz-Center for Agricultural Landscape Research, Eberswalder Str. 84, Müncheberg, Berlin, 15374, Germany

**Keywords:** *Anopheles atroparvus*, *Anopheles daciae*, *Anopheles maculipennis*, *Anopheles messeae*, Cytochrome c oxidase subunit 1, Germany, Internal transcribed spacer 2, Maculipennis group, Vector

## Abstract

**Background:**

*Anopheles daciae*, a newly described member of the Maculipennis group, was recently reported from western, southern and eastern Europe. Before its recognition, it had commonly been listed under the name of *An. messeae*, due to its extreme morphological and genetic similarities. As the sibling species of the Maculipennis group are known to differ in their vector competences for malaria parasites and other pathogens, the occurrence of *An. daciae* in a given region might have an impact on the epidemiology of mosquito-borne diseases. Mosquito collections from different localities in Germany were therefore screened for *An. daciae*.

**Methods:**

Adult and immature Maculipennis group mosquitoes were collected between May 2011 and June 2012 at 23 different sites in eight federal states of Germany. A standard PCR assay was used to differentiate the previously known sibling species while the ITS2 rDNA of specimens preliminarily identified as *An. messeae*/*daciae* was sequenced and analysed for species-specific nucleotide differences.

**Results:**

Four hundred and seventy-seven *Anopheles* specimens were successively identified to Maculipennis group level by morphology and to species level by DNA-based methods. Four species of the Maculipennis group were registered: *An. messeae* (n = 384), *An. maculipennis* (n = 82), *An. daciae* (n = 10) and *An. atroparvus* (n = 1). *Anopheles daciae* occurred at four sites in three federal states of Germany, three of the sites being located in north-eastern Germany (federal states of Brandenburg and Saxony) while one collection site was situated in the northern Upper Rhine Valley in the federal state of Hesse, south-western Germany.

**Conclusions:**

The detection of *An. daciae* represents the first recognition of this species in Germany where it was found to occur in sympatry with *An. messeae* and *An. maculipennis*. As the collection sites were in both north-eastern and south-western parts of Germany, the species is probably even more widely distributed in Germany than demonstrated, albeit apparently with low population densities. Research is needed that confirms the species status of *An. daciae* and elucidates its vector competence as compared to *An. messeae* and the other species of the Maculipennis group, in order to optimize management of possible future outbreaks of diseases caused by pathogen transmission through Maculipennis group mosquitoes.

## Background

The recognition of sibling species within the Maculipennis group of the culicid genus *Anopheles* in the early 20^th^ century and of their different roles as vectors of malaria parasites was a historical milestone in malaria research [1,2]. It triggered in-depth research on the biology and ecology of the various geographical “*Anopheles maculipennis* races” and renewed taxonomic revisions of the genus *Anopheles*. Based on nucleotide sequence analysis of the nuclear ribosomal DNA (rDNA) second internal transcribed spacer (ITS2), Harbach [3] confirmed the monophyly of the Maculipennis group species in 2004 and divided them into three hierarchical systems of informal taxonomic subgroups (Maculipennis subgroup, Quadrimaculatus subgroup, Freeborni subgroup). According to this system, and under consideration of *An. artemievi*, a mosquito species described in 2005 [4], the Palaearctic members of the Maculipennis group, including *An. atroparvus*, *An. labranchiae*, *An. maculipennis*, *An. melanoon*, *An. messeae*, *An. sacharovi*, *An. artemievi*, *An. martinius* and *An. persiensis*, form the Maculipennis subgroup. The six first-mentioned species plus *An. beklemishevi* (Quadrimaculatus subgroup of the Maculipennis group) are distributed throughout Europe.

While egg morphology, larval and pupal chaetotaxy, ecological studies, hybridization experiments, zymotaxonomy and cytotaxonomy were mostly applied to identify sibling species in earlier culicid research, recent discoveries of cryptic species are often the results of DNA analyses [4,5]. Thus, Nicolescu *et al*. [6] described *An. daciae* as an additional previously unrecognized member of the Maculipennis group on the Black Sea coast in southern Romania by means of differences in the rDNA ITS2 sequence as compared to *An. messeae*, supported by mitochondrial DNA (mtDNA) cytochrome c oxidase subunit I (COI) sequence data and morphological peculiarities of the egg ornamentation. The larvae, pupae and adult stages of both species are indistinguishable, and both species have been found to be sympatric [6,7]. Prior to the description of *An. daciae*, a polymerase chain reaction (PCR) assay developed by Proft *et al.*[8] provided a reliable tool for the identification of the then known European Maculipennis group sibling species. Using that PCR assay, however, *An. daciae* is erroneously identified as *An. messeae* and remains unrecognized.

In the same year that Nicolescu *et al*. [6] described *An. daciae*, Di Luca *et al*. [9] published a comprehensive study on intraspecific polymorphisms in the ITS2 region of populations of *An. messeae* from Italy, The Netherlands, former Yugoslavia, Kazakhstan and England. The authors came up with five haplotypes each of which corresponded to a distinct geographical area. An additional investigation of an “*An. messeae*” population in southwest England [7] revealed that its ITS2 sequences were identical both to the England haplotype described by Di Luca *et al*. [9] and to the *An. daciae* type series from Romania [6]. A comparative analysis of partial mitochondrial COI gene sequences of mosquitoes collected by Di Luca *et al*. [9] in Kazakhstan and Italy with those of specimens of the *An. daciae* type series from Romania collected by Nicolescu* et al.*[6] suggests the occurrence of *An. daciae* in England and Romania as well [7].

While there are now eight species of the Maculipennis group known to occur in Europe, three of them have been described for Germany: *An. maculipennis*, *An. atroparvus* and *An. messeae*[10]. However, the recent findings of new members of the Maculipennis group in Europe, in particular of *An. daciae* in eastern, southern and western Europe (Romania, Italy, England), suggested that *An. daciae* might also be present in other European countries such as Germany. Specimens of the Maculipennis group from a German national mosquito monitoring programme identified as *An. messeae* by the PCR assay according to Proft *et al.*[8] were therefore analyzed with regard to their ITS2 DNA sequences.

## Methods

**Figure 1:**
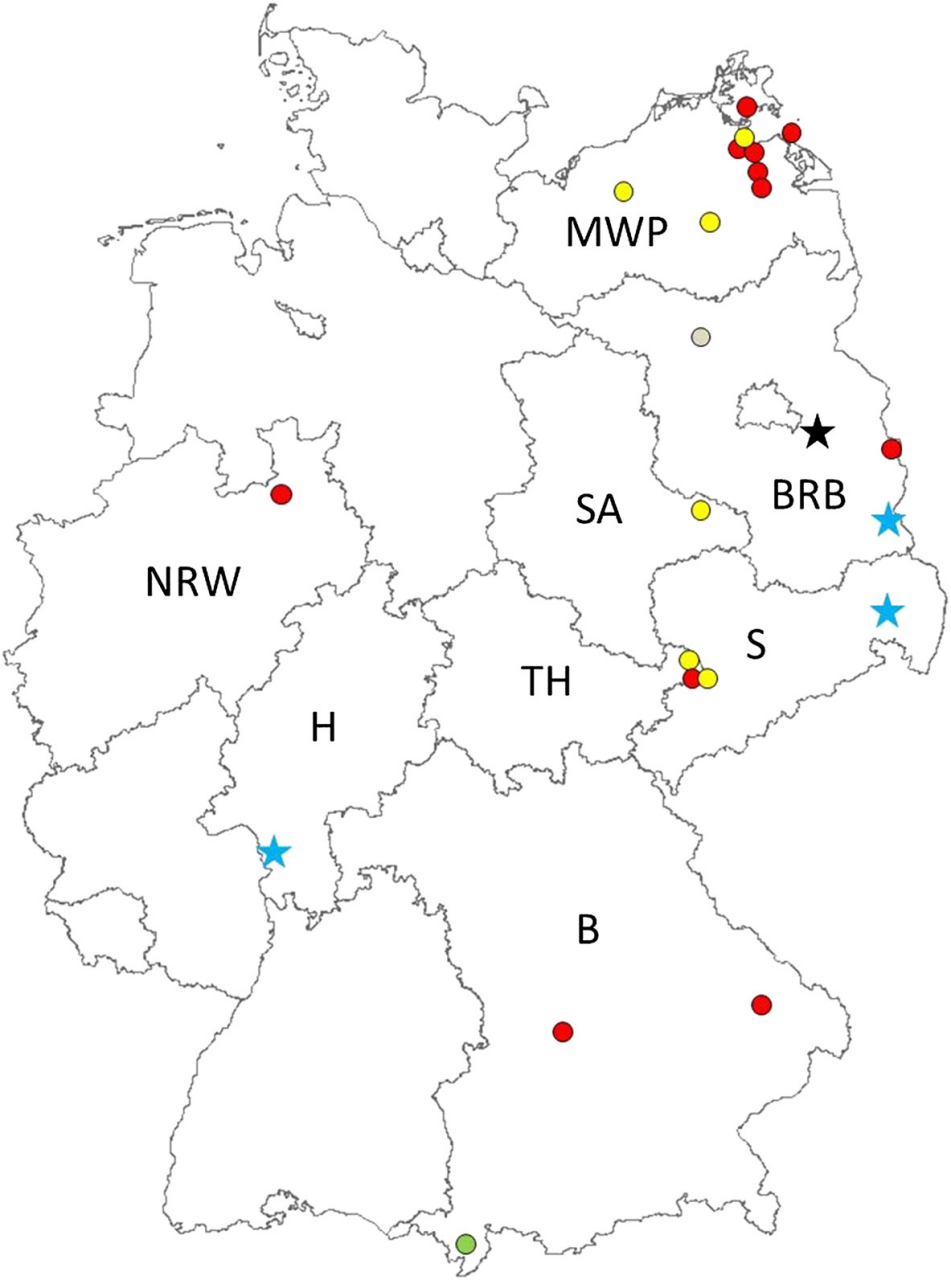
**Geographic locations of mosquito sampling sites and distribution of Maculipennis group species.** Asterisks and dots: sampling sites positive for mosquitoes of the Maculipennis group. Asterisk = *An. daciae* present. Colours: black = *An. daciae* and *An. maculipennis*; blue = *An. daciae, An. maculipennis* and *An. messeae*; red = *An. messeae*; green = *An. maculipennis*; yellow = *An. messeae* and *An. maculipennis*; grey = *An. maculipennis*, *An. messeae* and *An. atroparvus* present.

Mosquito specimens of the Maculipennis group were collected between May 2011 and June 2012 at 23 sites in eight federal states of Germany within the framework of mosquito monitoring activities (Table 1, Figure 1). Adult *Anopheles* specimens were caught by trapping and netting, as well as by hand collections from resting places in overwintering shelters and in animal stables during summer. Larvae and pupae were removed from their breeding sites and reared to adults for easier morphological identification which was done using the keys by Schaffner *et al.*[11] and Becker *et. al*[12]. Mosquitoes belonging to the Maculipennis group were further identified by a species-specific PCR assay [8] performed on DNA extracted from whole single specimens using the DNeasy Blood & Tissue Kit (Qiagen, Germany) and the NucleoSpin RNA Virus Kit (Macherey-Nagel, Germany) according to the instruction manuals. PCR products were fractionated on 1.5% agarose gels containing 0.5 μg/ml ethidium bromide and visualized under UV light. The ITS2 rDNA of specimens preliminarily identified as *An. messeae* was subsequently amplified using 5.8S and 28S primers published by Collins & Paskewitz [13] to generate DNA fragments of 435 bp each. For DNA sequencing, PCR products were cycled using the Big Dye Terminator v1.1 Cycle Sequencing Kit (Applied Biosystems, Germany). They were cleaned by means of SigmaSpin Sequencing Reaction Clean-Up columns (Sigma-Aldrich, Germany) and sequenced on a 3130 Genetic Analyzer (Applied Biosystems). Sequences were edited and aligned with published ITS2 sequences of *An. messeae* and *An. daciae* available in GenBank using CodonCode Aligner (CodonCode Corporation).

**Table 1:** Origin and species assignment of the Maculipennis group mosquitoes involved

**Federal state**	**Locality**	**No. identified**	***An. maculipennis***	***An. atroparvus***	***An. messeae***	***An. daciae***
Mecklenburg-Western Pomerania (MWP)	Boltenhagen	1	–	–	1	–
	Dummerstorf	2	1	–	1	–
	Greifswald	2	–	–	2	–
	Gristow	3	1	–	2	–
	Kargow	4	1	–	3	–
	Peendemuende	15	–	–	15	–
	Putbus	4	–	–	4	–
	Spantekow	2	–	–	2	–
	Tutow	13	–	–	13	–
Brandenburg (BRB)	Eisenhuettenstadt	2	–	–	2	–
	Maust	72	2	–	67	3
	Schoeneiche	16	15	–	–	1
	Zippelsfoerde	56	8	1	47	–
Saxony-Anhalt (SA)	Kropstaedt	61	36	–	25	–
North Rhine-Westphalia (NRW)	Bielefeld	2	–	–	2	–
Saxony (S)	Haselbach	99	3	–	96	–
	Ralbitz-Rosenthal	96	6	–	86	4
Thuringia (TH)	Windischleuba	4	–	–	4	–
	Zschaschelwitz	7	2	–	5	–
Hesse (H)	Trebur	5	1	–	2	2
Bavaria (B)	Deggendorf	1	–	–	1	–
	Agathazell	6	6	–	–	–
	Neuburg	4	–	–	4	–
**Total**		**477**	**82**	**1**	**384**	**10**

## Results and discussion

Four hundred and seventy-seven collected *Anopheles* specimens were assigned to the Maculipennis group according to morphological characters. Of these, ITS2 rDNA species-specific PCR according to Proft *et al*. [8] generated 394 *An. messeae*, 82 *An. maculipennis* and 1 *An. atroparvus*. While *An. messeae* and *An. maculipennis* have previously been shown to have a widespread distribution in Germany, the salt-tolerant species *An. atroparvus* mainly occurs in coastal marsh regions but has also been found in inland areas, although at much lower frequencies [14]. In total, “*An. messeae*” accounted for 80% of our *Anopheles* PCR identifications.

DNA sequence analysis of the ITS2 region of the “*An. messeae*” mosquitoes revealed five single nucleotide polymorphisms in ten specimens, nine females and a male (GenBank accession nos.: JX173885, JX416347-52, JX444557-59), identical to those defining *An. daciae* according to Nicolescu *et al.*[6]. Three of the females were hand-collected in August 2011 and June 2012 in a domesticated rabbit stall in Maust, Brandenburg, north-eastern Germany, close to the border with Poland. Four *An. daciae* females were sampled in June 2012 in a stable harbouring sheep in Ralbitz-Rosenthal, Saxony, and one male was caught in August 2011 in a rabbit stall in Schoeneiche, Brandenburg. The two remaining females were trapped by a BG-Sentinel mosquito trap (Biogents, Germany) in August and September 2011 in Trebur, Hesse. In all locations, either *An. messeae* or *An. maculipennis* or both were also shown to occur.

This is the first description of *An. daciae* for Germany. Considering known differences in vector competence and/or vectorial capacity for malaria parasites of different Maculipennis group species in the same geographic region and of the same species in different geographical areas, the status of *An. daciae* as a vector in Germany and elsewhere should be investigated. Such studies, however, should not remain restricted to malaria parasites but should include further pathogens since Maculipennis group sibling species have been shown to be infected in the field with Ťahyňa virus in Austria [15], West Nile virus in Portugal [16], Sindbis and Batai viruses in Germany [17,18], and *Dirofilaria immitis* and *Setaria labiatopapillosa* filaria in Italy [19,20].

Despite having followed the recent literature and having denominated *An. daciae* a species, the authors do not consider the evidence given for the species status of *An. daciae*, separate from *An. messeae*, as convincing and sufficient. There are three criteria on which the suggested species status of *An. daciae* is based, most importantly ITS2 rDNA sequence polymorphisms, with *An. daciae* being described as an ITS2 variant of *An. messeae* different at five positions out of 435 nucleotides. However, while investigating the intragenomic heterogeneity of the ITS2 region of geographically distinct *An. messeae* populations, Bezzhonova & Goryacheva [21] found that the *An. daciae* variant was just one out of various variants in peripheral populations of *An. messeae*, the other variants not being elevated to species status. Admittedly, the *An. daciae* variant was the only one found at more than one, geographically distinct location, which indicates that the genetic divergence is stable. In our ITS2 sequence analyses, the *An. daciae* ITS2 variant was the only one encountered in addition to the *An. messeae* variant.

A second criterion given by Nicolescu *et al*. [6] is the egg structure, which is considered different from that of *An. messeae*. The differences given, however, are minor and have not been shown to be statistically significant, i.e. to be outside the range of natural phenotypic variation within a species. In fact, such variation can be commonly observed in insect specimens of the same species including the Maculipennis group members [22].

Most ambiguous is the delimitation of *An. daciae* and *An. messeae* by means of unique polymorphisms in the COI gene, which, although used for species identification by barcoding, displays a certain degree of sequence variability [23]. While some COI sequence haplotypes are said to represent *An. daciae*[6], no data on intraspecific sequence divergence, either for *An. messeae* or for *An. daciae*, in contrast to interspecific divergence, have yet been published. Phylogenetic tree construction from GenBank COI sequences to check for clustering is not possible since it is not known without the corresponding ITS2 sequences whether sequence entries running under the name of *An. messeae* must actually be assigned to the *An. messeae* or to the *An. daciae* variant. Studies on correlated COI and ITS2 sequence analyses have therefore been initiated. Preliminary analyses of COI sequences of *An. messeae* specimens identified in our lab by ITS2 sequences, as compared to *An. daciae* COI sequences presented by Nicolescu *et al.*[6], have shown an identical haplotype. In support of such studies, the ecological and/or physiological features of *An. daciae* should be studied.

## Conclusion

To resolve the species status of *An. daciae*, it is necessary to correlate its genetic variant to well-defined biological characteristics and to carry out crossing experiments. Irrespective of that, vector competences and characteristics different from those of *An. messeae* are conceivable in the *An. daciae* variant that could, for instance, lead to, and explain, differences in the epidemiology of mosquito-borne diseases whose agents are transmitted by species of the Maculipennis group. Therefore, the exact geographical distribution and the vector status of *An. daciae* should be examined more carefully.

## Abbreviations

COI: Cytochrome oxidase subunit I; ITS2: Internal transcribed spacer 2; mtDNA: Mitochondrial DNA; rDNA: Ribosomal DNA.

## Misc

Mandy Kronefeld, Doreen Werner, Helge Kampen contributed equally to this work.

## Competing interests

The authors declare that they have no competing interests.

## Authors’ contributions

As part of her doctoral thesis, MK collected the mosquitoes, conducted a significant part of the mosquito identification and contributed to DNA sequencing, data analysis and drafting of the manuscript. MD and DZ participated in the mosquito collections and morphological identifications. DW designed the study, collected most of the mosquitoes, performed morphological identifications, and contributed to data analysis and manuscript drafting. HK designed the study, collected the mosquitoes, supervised and contributed to all aspects of laboratory work, and was involved in the data analysis and writing of the manuscript. All authors read and approved the final version of the manuscript.
